# DNA extraction of bacterial cells using a semi-automated filtration system

**DOI:** 10.1016/j.mex.2022.101785

**Published:** 2022-07-13

**Authors:** K.B. Hoorzook, T.G. Barnard

**Affiliations:** University of Johannesburg, South Africa

**Keywords:** Celite, Environmental water, *Escherichia coli*, Genomic and plasmid extraction, Guanidium thiocyanate, Membrane filtration, Semi-automated filtration system

## Abstract

The COVID-19 pandemic lockdown created problems with importing of commercial kits resulting in extended turnaround times for consumable deliveries. One way to circumvent this was to use an inexpensive optimized in-house method for DNA extraction from water.

• The DNA extraction methods were optimized on a 96-well plate using a semi-automated filtration system to increase the number of samples from 24 to 96 at a time in 2 h. The DNA extraction method optimizations included: (a) Guanidium thiocyanate method plus dilution series of celite to determine DNA binding capacity; (b) QIamp 96 Qiacube HT kit (Qiagen®); (c) Guanidium thiocyanate with the celite replaced with a binding buffer.

• The in-house DNA extraction methods and adapted in-house DNA extraction method were compared to QIamp 96 Qiacube HT kit (Qiagen®), which is used on a 96-well semi-automated filtration system. The results showed maximum capacity of the 96-well filter plates was 400 μℓ broth (OD_600_ = 0.45 = 3.6 × 10^8^ cells/mℓ) before the 96-well filters blocked.

• When the methods were compared, there was no significant difference between the in-house DNA extraction method with 1:420 celite dilution (*P*-value = 0.126) and the adapted in-house method with binding buffer (*P*-value = 0.298) DNA yield or amplification of PCR products.

## Graphical abstract

**Figure:**
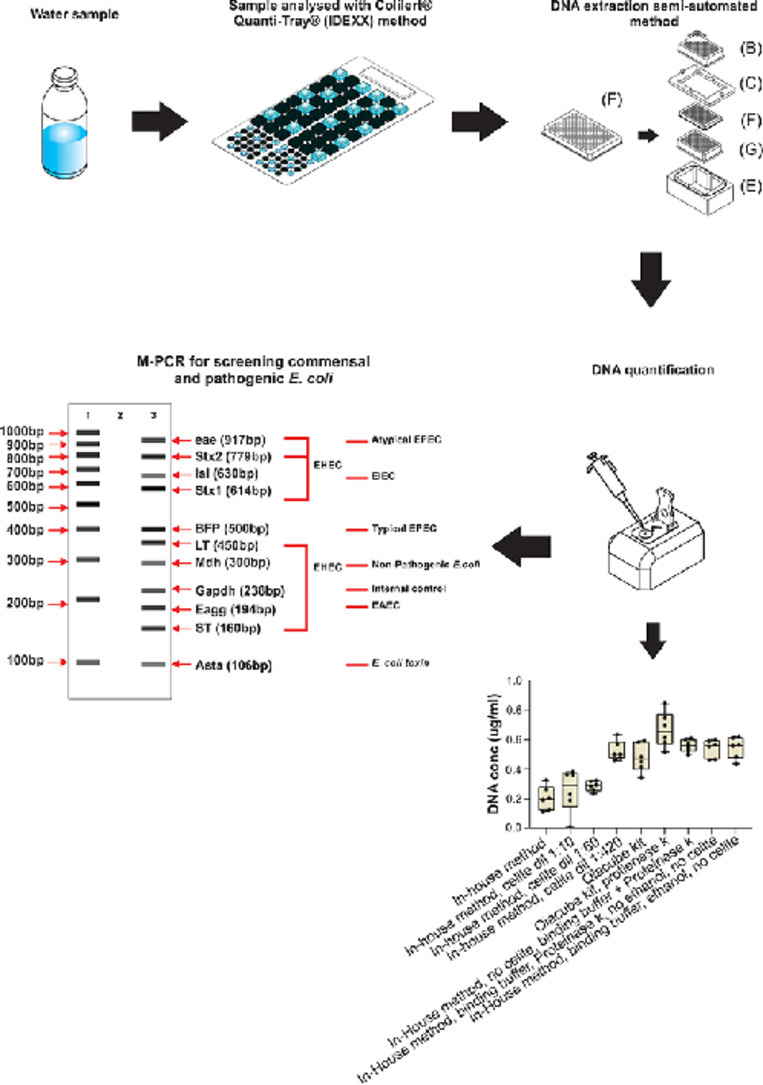

## Specifications table

**Table:** 

Subject Area;	Biochemistry, Genetics and Molecular Biology
More specific subject area;	*Semi-automated DNA extraction*
Method name;	*Semi-automated DNA extraction method*
Name and reference of original method;	(1) Boom R, Sol CJA, Salimans MMM, Jansen CL, Wertheim-Van Dillen PME and Van Der Noordaa J. (1990) Rapid and Simple Method for Purification of Nucleic Acids. Journal of Clinical Microbiology, 28: 495–503. (2) Omar KB, Potgieter N, Barnard TG. Development of a rapid screening method for the detection of pathogenic *E. coli* using a combination of Colilert® Quanti-Trays/2000 and PCR. Water Science and Technology: Water Supply. 2010;10 (*1*):7–13.
Resource availability;	*Logbooks, UJ Repository*

## Methodology

### Growth and maintenance of bacterial strains

A commensal non-pathogenic *E. coli* (ComEC) strain (ATCC 25922) was used in this study for the loading capacity experiment. The strain was cultured from frozen glycerol stock on Plate Count Agar (PCA) (Oxoid, UK) and incubated under aerobic conditions at 37 °C for 16 h. Single colonies were enriched in 100 mℓ sterile distilled water using the Colilert® Quanti-Tray® (IDEXX) and incubated under aerobic conditions at 37 °C for 16 h. After incubation a total of 20 mℓ of the broth was removed from 10 wells and aliquoted into a 50 mℓ Falcon tube. The absorbance reading at 600 nm (OD_600_) of this broth was 0.45 correlating to 3.6 × 10^8^ cells/mℓ.

One environmental water sample (river) was tested where 100 mℓ of a river water sample was enriched in the Colilert® Quanti-Tray® (IDEXX) and incubated under aerobic conditions at 37 °C for 16 h. After incubation a total of 20 mℓ of the broth was removed from 10 wells and aliquoted into a 50 mℓ Falcon tube. The OD_600_ of the sample was 0.49, i.e. equivalent to 3.92 × 10^8^ cells/mℓ.

### Buffer composition and preparation

The preparation of the celite, lysis buffer and washing buffer used for the in-house DNA extraction and adapted in-house DNA extraction method was as follows:

#### Celite

Celite (Sigma Aldrich) was prepared by suspending 10 g in 50 mℓ distilled water and adding 500 μℓ hydrochloric acid (HCl) (32% w/v) to the solution. Thereafter it was sterilised for 15 min at 121 °C and the bottle wrapped in aluminium foil and refrigerated (stable for 3 weeks at room temperature) (original solution) [1].

Celite dilution series is as follow:

(a) 1:10 – 50 µℓ (original celite) + 550 µℓ sterile distilled water.
(b) 1:60 – 10 µℓ (original celite) + 590 µℓ sterile distilled water.
(c) 1:420 – 1 µℓ (original celite) + 419 µℓ sterile distilled water.

#### Lysis buffer

Lysis buffer was prepared to a final concentration of 5 M Guanidium thiocyanate, by dissolving 120 g guanidinium thiocyanate (GuSCN) (Sigma Aldrich) in 100 mℓ of 0.1 M hydroxymethyl amino methane-hydrochloric acid (Tris-HCl) (pH 6.4) (Sigma Aldrich) in a 500 mℓ Schott bottle. The bottle was heated to 60 °C to dissolve the GuSCN. After heating, 22 mℓ of a 0.2 M ethylenediamine tetra-acetate (EDTA) (pH 8.0) with 2.6 mℓ triton X-100 (Sigma Aldrich) solution was added to the suspension. The suspension was mixed and dispensed into 50 mℓ Eppendorf tubes and 0.5 mℓ of celite suspension was added to remove any contaminating DNA from the buffer. The final solution was left to stand for at least 1 h at room temperature with sporadic mixing. The celite was pelleted by centrifugation at 3000 rpm for 10 min (NeoFuge-15R, Heal Force, Vacutec®) and the supernatant was transferred into sterile 50 mℓ Eppendorf tubes wrapped in aluminium foil (stable 3 weeks at room temperature).

#### Binding buffer

Binding buffer was prepared to a final concentration of 5 M Guanidine hydrochloride and 40% isopropanol. The bottle was heated to 60 °C to dissolve the Guanidine hydrochloride. The solution was filter sterilised using 25 mℓ syringe (Laboratory and Scientific Equipment) and 0.2 µm syringe filter (Laboratory and Scientific Equipment), stable at room temperature.

#### Wash buffer

Wash buffer was prepared to a final concentration of 5 M Guanidium thiocyanate, up by dissolving 120 g GuSCN and 100 mℓ of 0.1 M Tris-HCl (pH 6.4) in a 500 mℓ Schott bottle, heated to 60 °C to dissolve the GuSCN and dispensed into 50 mℓ Eppendorf tubes. Thereafter, 0.5 mℓ celite suspension was added to each tube to remove contaminating DNA from the buffer as described above.

#### Wash ethanol

A 70% (v/v) ethanol solution was prepared with sterile distilled water.

### Multiplex polymerase chain reaction (PCR)

The extracted DNA was amplified with a single multiplex PCR (m-PCR) targeting 11 *E. coli* genes from commensal and pathogenic *E. coli* to visually compare the DNA amplification efficiency of the DNA extracted with the various methods when viewed on the agarose gels.

The extracted DNA were amplified in a 20 µℓ reaction mixture containing 10 µℓ of the 2 x Qiagen® m-PCR master mix (Hotstart Taq DNA polymerase, 10 x buffer, 2 mM MgCl2 and dNTP mix), 1 µℓ 5 x Q-solution, 4.5 µℓ of PCR grade water, 2 µℓ l MgCl2, 2 µℓ of template DNA and 0.5 µℓ of the primer mix [0.1 μM of *mdh* and *lt*-1 primers (F and R), 0.2 μM of *ial, gapdh, eagg, astA*, and *bfpA* primers (F and R), 0.3 μM of *eaeA* and *stx2* primers (F and R), 0.5 μM of *stx1* and *st*-a primers (F and R)] [2].

All virulence genes were amplified in a BIO-RAD® T100™ Thermal Mycycler under the following conditions: enzyme activation at 95 °C for 15 min, 35 cycles of DNA denaturation at 94 °C for 45 s, annealing at 55 °C for 45 s, elongation at 68 °C for 2 min with a final elongation step at 72 °C for 5 min. For the negative control reaction mixture, the template DNA was replaced with sterile PCR grade water and the positive control reaction contained the combined extracted plasmids from the transformed *E. coli* strains [2].

Amplified products were separated according to their molecular size on a horizontal agarose gel slab [2.5% (w/v)] containing ethidium bromide (0.5 μg/mℓ) using TAE buffer. Electrophoresis was conducted at 80–100 Volts for 30–50 min and viewed under UV light (Gene Genius Bio Imaging System, Vacutec®). The relevant sizes of the DNA fragments were estimated by comparing their electrophoretic mobility to that of a standard 100 bp marker (Quick-Load®, New England BioLabs®).

### In-house DNA extraction method

The DNA extraction method used as starting point for the experiments was based on the method reported by Omar et al. [3] who used a modification of the Boom et al. [1] protocols. The in-house DNA extraction method used guanidium thiocyanate to lyse the bacterial cells and silicon dioxide-based material (such as celite) to bind genomic and plasmid DNA [1]. In the 96-well semi-automated system, the celite added a second DNA binding step to the 96-well filter plate to increase the DNA yield.

**Fig. 1:**
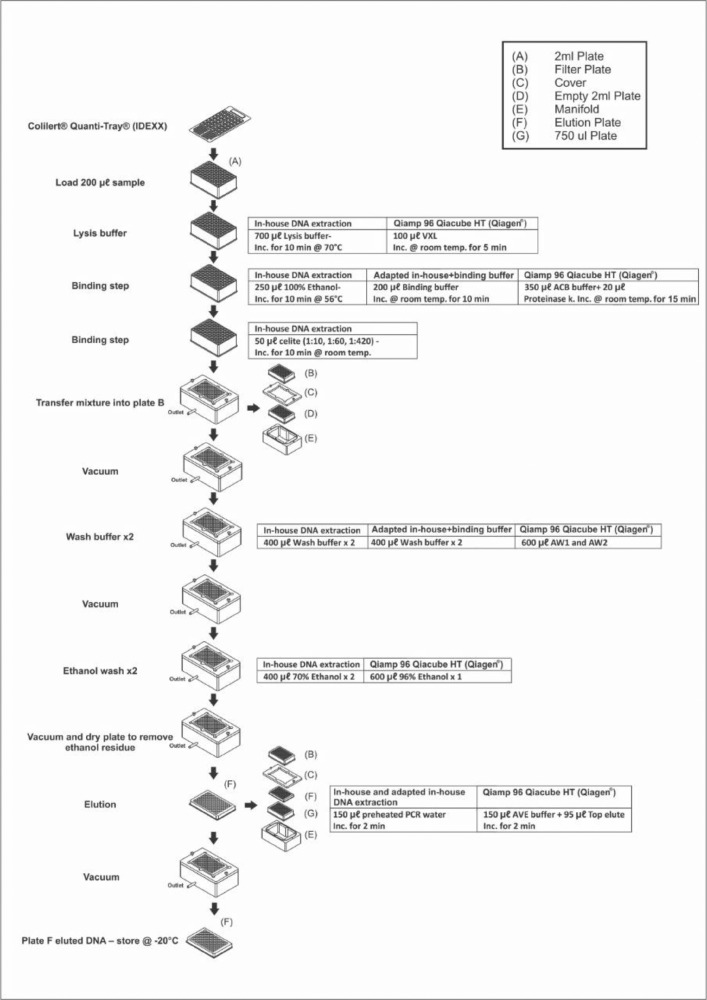
Summary of the in-house DNA extraction showing the changes made to the 96-well DNA extraction methods [Adapted from Delair [4]].

Briefly the methodology is as follows (Fig. 1):

Colilert® Quanti-Tray® broth (200 μℓ) was aliquoted into each of the 96-well preparation plate (2 mℓ V-bottom, Greiner bio-one (Cat log no. 780285-914)). To this 700 μℓ of lysis buffer was added and incubated at 70 °C for 10 min. Thereafter, 250 μℓ of 100% ethanol was added and incubated at 56 °C for 10 min. Then, 50 μℓ of celite was added to the mixture and incubated for 10 min at room temperature, a series of celite dilutions was prepared (Fig. 1). The suspension was transferred into the 96-well filter plate (Whatman, Merck Life Science) and vacuum filtered to remove supernatant. The celite pellet was subsequently washed twice with 400 µℓ of wash buffer followed by vacuum filtration to remove supernatant. Two more wash steps with 400 µℓ of 70% (v/v) ethanol followed this. The pellet was vacuum dried before an elution plate (Eppendorf, Merck Life Science) was placed below the filter plate. Thereafter, 150 μℓ of preheated at 56 °C PCR water was added, the pellet was re-suspended and waited for 2 min before vacuum filtration to elute the DNA.

### Adapted in-house DNA extraction method

The adaptation of the in-house DNA extraction method was to replace celite solution with a binding buffer. The protocol was thus using 200 μℓ Colilert® Quanti-Tray® broth to which 700 μℓ of lysis buffer was added and incubated at 70 °C for 10 min. Thereafter, 200 μℓ of binding buffer was added and incubated for 10 min at room temperature. The suspension was transferred into the semi-automated 96-well filter plate (Whatman, Merck Life Science) and vacuum filtered to remove supernatant. The remaining protocol was followed as described above.

### QIAamp 96 DNA QIAcube HT kit (Qiagen®) commercial kit

Colilert® Quanti-Tray® broth 200 μℓ was aliquoted into each of the 96-well preparation plate. To this 100 μℓ of VXL as instructed by the manufacturer was added and incubated for 5 min at room temperature. Thereafter, added 20 μℓ proteinase K and incubated at room temperature for 15 min, then 350 μℓ of ACB buffer was added. The suspension was transferred into the 96-well filter plate and vacuum filtered to remove supernatant. Two wash steps with 600 µℓ of AW1 and AW2 buffer were added separately followed by vacuum filtration. Followed by 600 µℓ of 96% ethanol and vacuum filtration to remove supernatant. The pellet was vacuum dried before a 96-well elution plate was placed below the 96-well filter plate. Thereafter, 150 μℓ of PCR water preheated at 56 °C was added, the pellet re-suspended and waited for 2 min before vacuum filtration to elute the DNA.

### Experimental design

Originally, the in-house DNA extraction method was optimised to be used with manual spin columns adapted from Borodina et al. [5]. However, we wanted to adapt the in-house DNA extraction method [3] to be used on the semi-automated 96-well vacuum filtration system. To test the method on 96-well filter plates, factors such as filter blocking from celite, binding capacity of the filter (with and without the celite) and potential influence of proteinase K (used in the commercial kit) were tested. This was achieved by testing the following using one bacterial solution with each variable tested with six repeats:

(1) Original in-house DNA extraction method with undiluted celite.
(2) In-house DNA extraction method with a series of celite dilutions (1:10; 1:60; 1:420).
(3) Adapted in-house DNA extraction method (no celite but with ethanol and binding buffer).
(4) Adapted in-house DNA extraction method, celite and ethanol step removed.
(5) Adapted in-house DNA extraction method, celite and ethanol step removed but proteinase K added.
(6) QIAamp 96 DNA QIAcube HT DNA extraction kit with and without proteinase K.

This experiment used a river water sample enriched in the Colilert® Quanti-Tray® (IDEXX). The positive *E. coli* wells were used for subsequent experiments.

From the results obtained, a question arose regarding the loading capacity on the filters. A dilution series (1 ml, 800 µℓ, 600 µℓ, 400 µℓ and 200 µℓ) of the bacterial suspension removed from the Colilert® Quanti-tray® system to determine the concentration of cells allowed to be filtered before blocking the vacuum filtration system. This experiment was only performed in triplicate using the in-house DNA extraction method with 1:420 celite dilution and the adapted in-house DNA extraction method, with no celite and ethanol.

### Statistical analysis

The extracted DNA was quantified using Qubit™ fluorometer (Invitrogen USA) with the Quanti-It kit and reported as µg/mℓ as specified by the manufacturer. Statistical analysis was performed using Graphpad Prism® version 9 and IBM SPSS statistics version 28. The underlying normality (Shapiro-Wilk test) and homogeneity of variances (Levine's test) were tested to allow for further analysis for one-way ANOVA and Dunnett T3 Post-hoc parametric tests. The non-parametric test was used to check for any contradictions to the parametric tests, because there were less than 30 observations per sample. The Kruskal-Wallis tests were used to see if there were significant differences in the mean scores on the dependent variables across the groups. This test is an alternative to one-way ANOVA. The Mann-Witney U test was used to find out where these differences lie. This test is an alternative to post-hoc test.

## Results and discussion

Owing to lockdown regulations and difficulties experienced in importing commercial DNA extraction kits for the 96-well semi-automated system, we wanted to optimize the manual in-house DNA extraction method [3] and adapt it for the semi-automated 96-well vacuum filtration system for high throughput of analysis, additionally determine how comparable the in-house DNA extraction method is to commercially available 96-well DNA extraction kits [4]. Currently, with the manual in-house DNA extraction method, 24 samples can only be analyzed at a time, while with the semi-automated system, 93 samples plus 3 controls can be analyzed.

**Fig. 2:**
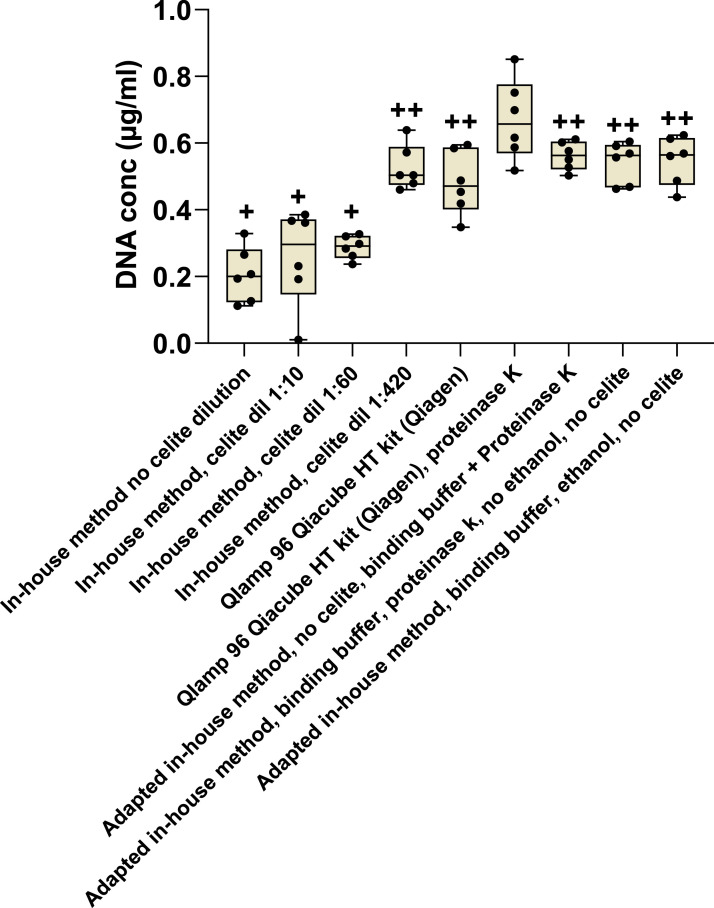
Box-Whisker plot comparing mean DNA yield obtained with the nine DNA extraction methods. Methods were compared to the to QIAamp 96 DNA QIAcube HT DNA extraction kit with proteinase K (Qiagen®) and statistically significant differences (*p* ≤ 0.05) are indicated by “+” with non-significant difference indicated with “++”.

**Fig. 3:**
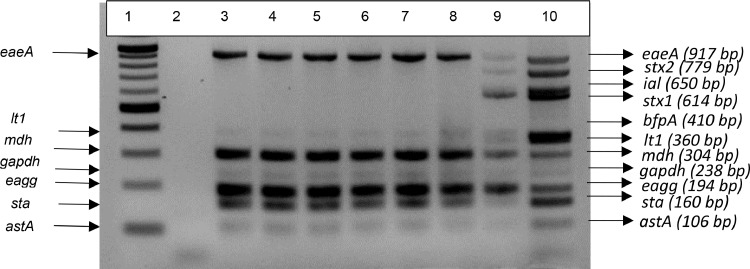
Example of agarose gel picture to illustrate quality of PCR amplified genes from the DNA extracted from the enriched river sample with the various methods. Shown in the gel is the 100 bp DNA ladder (lane 1), Negative control (NTC; lane 2), in-house DNA extraction method, celite 1:420 dil. (Lane3), QIAamp 96 DNA QIAcube HT DNA extraction kit (Qiagen®) (Lane 4), QIAamp 96 DNA QIAcube HT DNA extraction kit (Qiagen®) with proteinase K (Lane 5), adapted in-house method, with binding buffer and proteinase K and no celite (Lane 6), Adapted in-house method, with binding buffer and proteinase K, no celite and no ethanol (Lane 7), adapted in-house method, with binding buffer, no celite, no ethanol and no proteinase K (Lane 8), *E. coli* DNA ladder (Lane 9), *E. coli* positive control PCR (Lane 10).

The results for comparison of the three methods: (A) In-house DNA extraction method [3]; (B) Adapted in-house DNA extraction method, i.e. removal of celite and ethanol and an inclusion of the binding buffer; (C) Commercial DNA extraction method (Qiagen®) and the series of experiments conducted using the three methods from the enriched river water sample are shown in Fig. 2. The agarose gel pictures for the PCR from the enriched river water sample are shown in Fig. 3.

The river water sample was an environmental water sample and could contain the presence of commensal and pathogenic *E. coli* genes in a complex mixture. The 11-gene *E. coli* m-PCR confirmed that a variety of these genes could be successfully amplified from an environmental sample showing the presence of atypical Enteropathogenic *E. coli* (aEPEC), Enterotoxigenic *E. coli* (ETEC), Enteroaggregative *E. coli* (EAEC) and the *E. coli* toxin in the river water sample. The *mdh* is a malate dehydrogenase gene used as a control to confirm microbiology results in case no pathogenic genes were tested. No false positives or inhibition were indicated in the m-PCR as the external control (*gapdh*) was detected in the samples (Fig. 3).

The nine methods (Fig. 2) were statistically analyzed for the DNA yield as described in Section 1.8. The underlying normality (Shapiro-Wilk test) and homogeneity of variance (Levine's test) were firstly tested because of the small data set. The homogeneity of variance assumptions was not violated, which allowed for further one-way ANOVA analysis (Table 1). In normality the assumptions were not violated; however, non-parametric tests were used to check for any contradictions on the results provided by the parametric tests. From the analysis, non-parametric tests did not contradict the results (Table 1).

**Table 1:** Statistical comparison from the enriched river water sample between 96-well in-house DNA extraction method and adapted in-house DNA extraction method versus QIAamp 96 DNA QIAcube HT DNA extraction kit with proteinase K (Qiagen®).

DNA extraction methods	Mean (µg/mℓ)	Std. Dev	Std. error	*P*-value	CV
In-house DNA extraction	0.206	0.083	0.034	< 0.001 *	41%
In-house celite dil. 1:10	0.258	0.145	0.59	< 0.001 *	56%
In-house celite dil. 1:60	0.288	0.034	0.014	< 0.001 *	12%
In-house celite dil. 1:420	0.527	0.067	0,027	0.126	13%
Adapted in-house, no celite, binding buffer+proteinase K	0.562	0.043	0.17	0.445	8%
Adapted in-house, no ethanol and no celite, binding buffer+proteinase K	0.542	0.062	0.025	0.238	11%
Adapted in-house, no Celite no proteinase K, binding buffer and ethanol	0.549	0.073	0.030	0.298	13%
QIAamp 96 DNA QIAcube HT DNA extraction kit (Qiagen®)	0.481	0.096	0.039	0.013	20%
QIAamp 96 DNA QIAcube HT DNA extraction kit (Qiagen®), proteinase K	0.670	0.121	0.049	–	18%

NB: *P* ≥ 0.05 non-statistically different; *P* ≤ 0.05 statistically different*.

When the in-house DNA extraction method and the adapted in-house DNA extraction method were compared to the commercial testing kit (Qiagen®) (Table 1), using one-way ANOVA and Dunnett T3 post-hoc test to determine in which pairs of methods significant differences lie. Highly significant interactions between the in-house DNA extraction method and QIAamp 96 DNA QIAcube HT DNA extraction kit with proteinase K (Qiagen®), except for celite dilution 1:420. This is expected since the celite with no dilution, dilution of 1:10 and 1:60 blocked the wells. No significant interactions between QIAamp 96 DNA QIAcube HT DNA extraction kit with and without proteinase K (Qiagen®); in-house celite dilution 1:420 and the three adapted in-house DNA extraction methods (Table 1). Furthermore, the robust test of equality of means was done, using Brown-Forsythe, thus further confirming the F distribution between the groups (20.683) is the same as the one-way ANOVA therefore, no contradictions. Adams [6] stated that data should be presented in a way which would allow the reader to observe the amount of variation inherent to the experiment, for example, mean, standard deviation (SD) and confidence intervals. Biological systems are subjected to variation and experimental imprecision and some statistics can reveal differences that are not otherwise discernible [7]. The coefficient of variation (CV) was used to measure intra-assay reproducibility from well to well and inter-assay variation from assay to assay, the smaller the CV the better the assay. Therefore, the CV was included for these analyses. The SD and CV from Table 1 indicate that the in-house DNA extraction method with celite dilution 1:420 and the three adapted in-house DNA extraction methods were lower than the QIAamp 96 DNA QIAcube HT DNA extraction kit with proteinase K (Qiagen®).

**Fig. 4:**
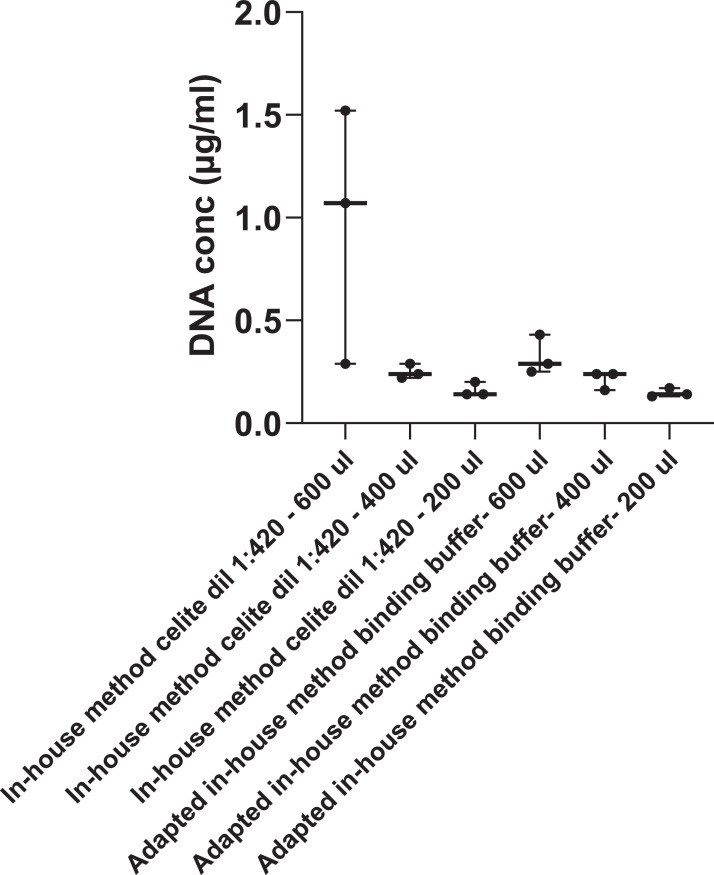
Box-Whisker plot illustrating the loading and binding capacity of the DNA extraction methods.

To determine the loading capacity, a dilution series (1 mℓ, 800 µℓ, 600 µℓ, 400 µℓ and 200 µℓ) was removed from the Colilert® Quanti-tray® system to determine the concentration of cells allowed to be filtered before blocking the vacuum filtration system. Fig. 4 indicates the binding capacity for the DNA extraction methods.

The result illustrates that 200 µℓ (∼7.9 × 10^7^ cells) and 400 µℓ broth (∼1.4 × 10^8^ cells) filters through with no blockages and consistent yields are recovered. In conclusion, the in-house DNA extraction method with 1:420 celite dilution and the three adapted in-house DNA extraction with binding buffer method provided comparable DNA yields to the Qiagen® commercial 96-well DNA extraction kit.

The aim of this method was to develop an inexpensive DNA extraction method using 96-well plates, which is comparable to the available DNA extraction 96-well commercial kit. The method was developed for use with *E. coli* grown in the selective media of the Colilert® Quanti-Trays for PCR testing as shown in Fig. 3. In line with this, the DNA concentrations obtained (∼30–40 ng) are typical growth observed in the Colilert® Quanti-Trays and are sufficient for the ≤1 µg DNA required in a PCR reaction [8], [9]. Additional steps (such as homogenization and DNA precipitation) can be tested if other bacterial enrichments or samples will be used with this method to increase the DNA yield. Several studies have demonstrated variability in their DNA recovery efficiency. Studies have shown the DNA recovery efficiency never exceeded 50% and some of the commercial kits tested only recovered 2% on the input DNA [10], [11].

## Declaration of Competing Interest

The authors declare that they have no known competing financial interests or personal relationships that could have appeared to influence the work reported in this paper.

## Data Availability

Data will be made available on request.

## Appendix Supplementary materials

Image, application 1
