# Supplementary material for: DNA extraction of bacterial cells using a semi-automated filtration system
Source: MethodsX. 2022 Jul 13;9:101785. doi: 10.1016/j.mex.2022.101785 (PMC9277997; doi:10.1016/j.mex.2022.101785)
Supplement: Supplementary file 1 [file mmc1.docx]

**Supplementary material *and/or* Additional information:** *Additional supplementary material can be found at the UJ Repository.*

***Background***

Certain bacteria such as *Escherichia* coli (*E. coli*) are firstly pre-enriched, i.e. grown in an enrichment medium (buffered peptone water) for 24 h before DNA can be extracted and used for Polymerase Chain Reaction (PCR) assay [1]. A pre-enrichment step is incorporated to detect pathogens present in low numbers [2]. It also provides a practical approach to determine the presence /absence of bacteria in a sample as these methods are simple and do not require considerable expertise [2;3], like the Colilert^®^ Quanti-Tray^®^ method. For this method, you pour the water sample into the tray, add media, shake, seal, and incubate. Obtaining high quality DNA is critical for a successful PCR process [4;5]. Therefore, extraction methods used for DNA isolation are selected on their specificity [6]. Extraction protocols functions used three major processes, lysis of the cell, removal/separation of non-nucleic acid constituents and purification of the nucleic acid material [6]. Lysis of cells is achieved chemically by phenol/chloroform or Guanidium thiocyanate (GuSCN) or physically by glass beads. Guanidium thiocyanate is a compound that has two properties, which makes it useful for DNA purification. First, it denatures and dissolves all biochemicals other than the nucleic acids and can therefore be used to release DNA from virtually any type of tissue. Secondly, in the presence of this compound, DNA binds tightly to silica or celite particles. This provides an easy way of recovering the DNA from the denatured mix of biochemicals. What is also very important is that the DNA binding to the silica or celite particles is influenced by salt concentration – the more salt, the greater the binding potential. This is a major part of the silica method as the nucleic acid is bound to the silica and washed using solutions with a high salt concentration and then eluted using a solution with a low salt concentration such as TE buffer or distilled water [7]. GuSCN has shown to be a powerful agent in the purification and detection of both RNA and DNA because of its potential to lyse cells combined with its potential to inactivate nucleases [8]. Majority of non-commercial DNA extractions and commercial kits are based on this method. Several DNA extraction kits are designed for large-scale analysis on a 96-well plate integrated with a robotic handler. The QIAamp 96 DNA QIAcube HT DNA Extraction kit (QIAGEN^®^) from the automated 96-well robot system, was validated and implemented on a semi-automated system [3]. This would facilitate the same reliable results with reduced cost implications. The semi-automated system is a manifold, which houses the filter plate attached to a vacuum pump. Reagents are manually added to the filter plate and vacuumed through. Purified DNA is collected and used in the m-PCR [3]. This study implemented the semi-automated manifold system for large-scale analysis of water samples to upscale from 24 samples per run to 96 samples. To further reduce cost implications and importing DNA extraction kits, the in-house DNA extraction and adapted in-house DNA extraction method were compared to the commercial DNA extraction kit.

**References:**

1. [J. Sidhu, S. Toze, Human pathogens and their indicators in biosolids: a literature review, Environ. Int. 35 (1) (2009) 187–201.](http://refhub.elsevier.com/S2215-0161(22)00165-0/sbref0012)
2. [K. Smith, M.A. Diggle, S.C. Clarke, Comparison of commercial DNA extraction kits for extraction of bacterial genomic DNA from whole-blood samples, J. Clin. Microbiol. 41 (6) (2003) 2440–2443 1.](http://refhub.elsevier.com/S2215-0161(22)00165-0/sbref0013)
3. Delair, Zaakirah. Implementation of molecular methods for the detection and characterization of pathogenic Escherichia coli: industrial and routine monitoring applications. Master’s in Biotechnology. Unpublished: University of Johannesburg. Available from: [http://hdl.handle.net/10210/226624. 2017](http://hdl.handle.net/10210/226624.%202017).
4. Myint MS, Johnson YJ, Tablante NL, Heckert RA. The effect of pre-enrichment protocol on the sensitivity and specificity of PCR for detection of naturally contaminated Salmonella in raw poultry compared to conventional culture. Food Microbiology. 2006, 23(6).
5. Smith K, Diggle MA, Clarke SC. Comparison of commercial DNA extraction kits for extraction of bacterial genomic DNA from whole-blood samples. Journal of Clinical Microbiology. 2003, 1;41(6):2440-3.
6. Beneduce L, Beneduce L, Fiocco D, Spano G. Development of PCR-based molecular tools for the detection of emerging food-ad water-borne pathogenic bacteria Novel methods for water and foodborne pathogenic bacteria detection View project Development of PCR-based molecular tools for the detection of emerging food-ad water-borne pathogenic bacteria. 2007.
7. Brown TA. Gene cloning and DNA analysis. 6th edition. USA: Blackwell Science Ltd; 2010. p. 50-53.
8. Ali N, de Cássia R, Rampazzo P, Dias Tavares Costa A, Krieger MA. Current Nucleic Acid Extraction Methods and Their Implications to Point-of-Care Diagnostics. 2017, 9306564:1-17, doi: 10.1155/2017/9306564.
